# Supplementary material for: Electrochemical Determination of Hydroxyurea in a Complex Biological Matrix Using MoS2-Modified Electrodes and Chemometrics
Source: Biomedicines. 2020 Dec 24;9(1):6. doi: 10.3390/biomedicines9010006 (PMC7823617; doi:10.3390/biomedicines9010006)
Supplement: Supplementary file 1 [file biomedicines-09-00006-s001.pdf]

Supplementary Materials

Electrochemical Determination of Hydroxyurea in a Complex Biological Matrix Using MoS_2-Modified Electrodes and Chemometrics

Remi Cazelles [1], Rajendra P. Shukla [1], Russell E. Ware [2,3,4], Alexander A. Vinks [4,5] and Hadar Ben-Yoav [1]

[1] Nanobioelectronics Laboratory (NBEL), Department of Biomedical Engineering and Ilse Katz Institute of Nanoscale Science and Technology, Ben-Gurion University of the Negev, Beer-Sheva 8410501, Israel.

[2] Division of Pediatric Hematology, Cincinnati Children's Hospital Medical Center, Cincinnati OH 45267, United States.

[3] Global Health Center, Cincinnati Children's Hospital Medical Center, Cincinnati OH 45267, United States.

[4] Division of Clinical Pharmacology, Cincinnati Children's Hospital Medical Center, Cincinnati OH 45267, United States.

[5] Department of Pediatrics, College of Medicine, University of Cincinnati, Cincinnati OH 45267, United States.

Table S1: The composition of our simulated serum consists of a list of 28 interfering molecules with a precise concentration (taken as the upper biological concentration limit) [1]. It comprises the commonly encountered redox active molecules uric acid (UA) and ascorbic acid (AA), known to interfere drastically with electrochemical measurements [2], as well as less prominent organic acidic molecule (red), amino acids (green), and ions (blue). Specifically, the simulated serum contains 0.6 nM dopamine (dilution 1:1000 v/v from 0.6 mM stock solution), 110 µM ascorbic acid (19.9 mg), 400 µM uric acid (67.2 mg), 12 µM oxalic acid (1.1 mg), 400 µM tri-sodium citrate (645,1 mg), 9 mM urea (540.5 mg), 1 mM ATP (551.1 mg), 170 µM acetone (12.6 mL), 58 µM methanol (2.3 mL), 670 µM L-glutamine (97.9 mg), 63 µM L-cysteine (7.6 mg), 6.1 mM D-glucose (1.1 g), 276 µM L-valine (32.3 mg), 38 µM L-methionine (5.7 mg), 217 µM L-lysine (31.7 mg), 2.4 mM L-lactic acid (216 mg), 150 µM L-Glutamic acid (22.1 mg), 407 µM L-Alanine (36.2 mg), 140 µM L-arginine (24.5 mg), 282 µM Glycine (21.2 mg), 800 µM magnesium sulfate (96.3 mg), 25 mM ammonium carbonate (2.4 g), 2.5 mM calcium chloride (277 mg), 9 mM iron sulfate (1.36 g), 10 mM di-sodium phosphate (1.42 g), 4.5 mM potassium chloride (335 mg), and 144 mM sodium chlorine (8.18 g instead of 8.41 g to compensate for chlorine ions already added as counter ions with previous compounds). Care must be taken to specifically choose salts whose counter ion concentrations are integrated into the overall concentration, calculating each of the simulated serum

components. After its preparation, the simulated serum can be stored at 4°C up to 1 month unless visible signs of bacterial contamination appear.

molecules	molecular weight (g/mol)	Concentration (M)	Concentration (mg/L)
Dopamine	153.18	6.00E-10	9.19E-05
Ascorbic acid	176.12	1.13E-04	19.90
Uric acid	168	4.00E-04	67.24
oxalic acid	90.03	1.20E-05	1.08
Tri sodiumm citrate	258.06	4.00E-04	103.22
Urea	60.06	9.00E-03	540.54
ATP	551.14	3.00E-03	1653.42
Acetone	58.08	1.70E-04	9.87
Methanol	32.04	5.80E-05	1.86
L-glutamine	146.14	6.70E-04	97.91
L-cysteine	121.16	6.30E-05	7.63
D-glucose	180	6.10E-03	1098.95
L-valine	117,151	2.76E-04	32333.68
L-methionine	149.21	3.80E-05	5.67
L-lysine	146.19	2.17E-04	31.72
L-lactic acid	90.08	2.40E-03	216.19
L-Glutamic acid,	147.13	1.50E-04	22.07
L-Alanine,	89.09	4.07E-04	36.26
L-arginine	174.2	1.41E-04	24.54
Glycine	75	2.82E-04	21.17
Magnesium sulfate	120	8.00E-04	96.29
ammonium corbonate	96.09	2.50E-02	2402.25
Calcium chloride	110.98	2.50E-03	277.45
Iron sulfate	152	9.00E-03	1367.17
sulfate (SO4 2-)	96.06	5.00E-04	48.03
Phosphate (di sodium)	142	1.00E-01	14200.00
potassium (chloride)	75	4.50E-03	335.48
chlorine (Na)	58	1.10E-01	6428.73
sodium	23	1.44E-01	3312.00

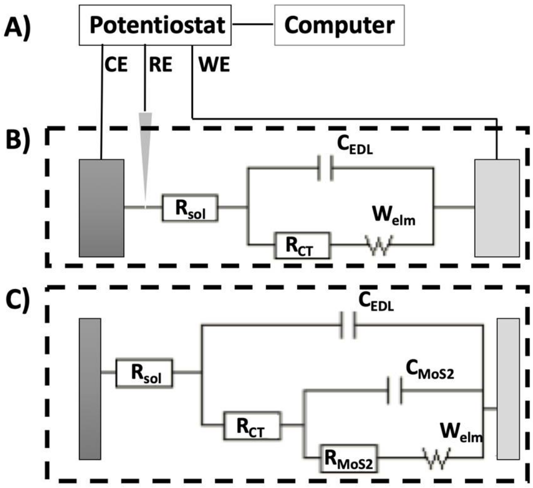

Figure S1: Electrochemical impedance spectroscopy (EIS) analysis. We used a three-electrode setup (A) and a solution containing a ferro/ferricyanide solution (5 mM) to acquire the EIS data. The EIS measurements were recorded at an open circuit potential (0.20 ± 0.05 $V_{Ag/AgCl}$) from 10 kHz to 0.1 Hz with 8 points per decade and with a sine-wave amplitude of 12 mV. The fitting of the EIS spectra was done via BioLogic EC-Lab software; two different equivalent electronic circuits were used. The first Randles Sevick equivalent electronic circuit used to fit the data acquired with the bare electrode (B) comprises the solution resistance (R_{sol}), the resistance to charge transfer (R_{CT}), the capacitance of the gold electrical double layer (C_{EDL}), and a Warburg element. The circuit used to fit the data acquired with the MoS_2-modified electrodes (C) comprises a supplementary resistance (R_{MoS2}) and capacitance (C_{MoS2}) corresponding to the electrodeposited material.

(A)

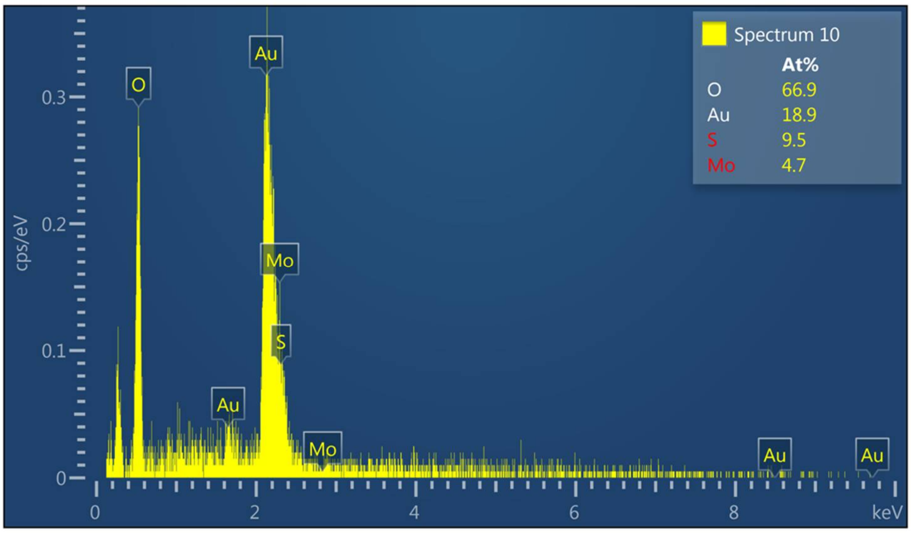

(B) **(C)**

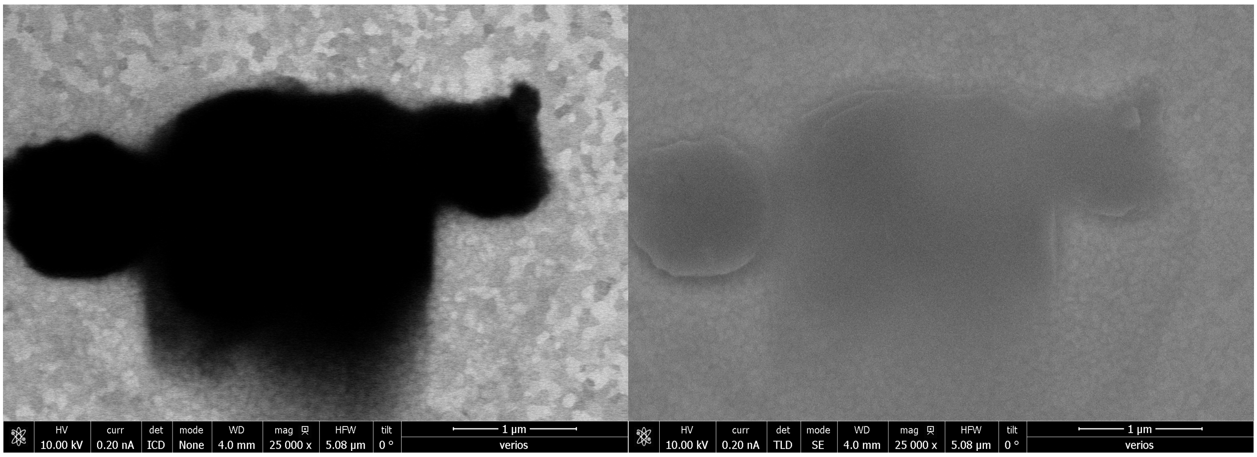

Figure S2: Energy-dispersive X-ray spectroscopy analysis (A) shows the presence of MoS_2 ($Mo_1S_{2.02}$). The Scanning Electron Microscope (SEM, Quanta 200, FEI) is coupled to an energy dispersive X-Ray spectroscopy instrument (Ultim®Max, Oxford Instruments). For the SEM images, we used an acceleration voltage of 10 kV and a current of 0.2 nA at a working distance of 4 mm. We used a back-scattered electron (A) and a secondary electron (B) detector.

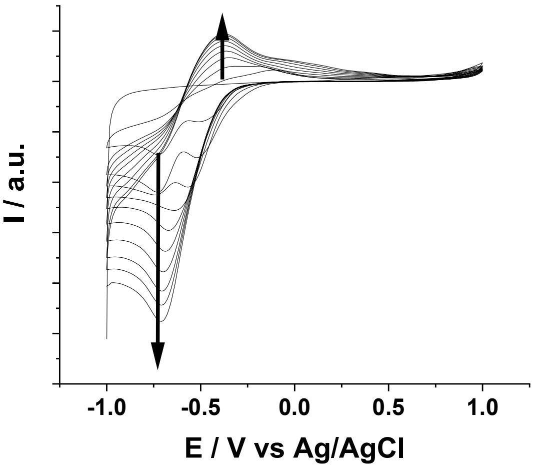

Figure S3: Representative cyclic voltammograms resulting from the electro-deposition of MoS_2 in the potential range of -1 < $V_{Ag/AgCl}$< +1. The electrolyte is composed of a solid powder containing nanoflakes of MoS_2 (GLMSW0A1, ACS Material LLC), dispersed by sonication at T = 25 C for 15 minutes in a 0.1 M H_2SO_4 solution at a concentration of 10 g L^{-1}. The cyclic voltammogram was recorded at 50 mV s^{-1} at the potential range of -1 < $V_{Ag/AgCl}$< +1; 10 cycles were recorded.

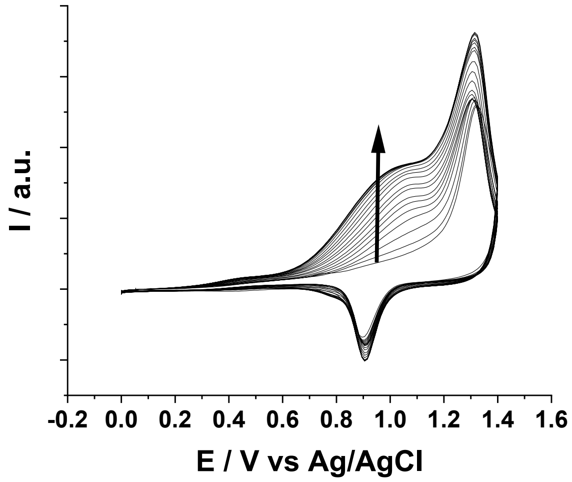

Figure S4: Representative cyclic voltammograms resulting from the electro-deposition of MoS_2 in the potential range of 0 < $V_{Ag/AgCl}$< +1.4. The electrolyte is composed of a solid powder containing nanoflakes of MoS_2 (GLMSW0A1, ACS Material LLC), dispersed by sonication at T = 25° C for 15 minutes in a 0.1 M H_2SO_4 solution and at a concentration of 10 g L^{-1}. The cyclic voltammogram is recorded at 50 mV s^{-1} in the potential range of 0 < $V_{Ag/AgCl}$< +1.4; 10 cycles are recorded.

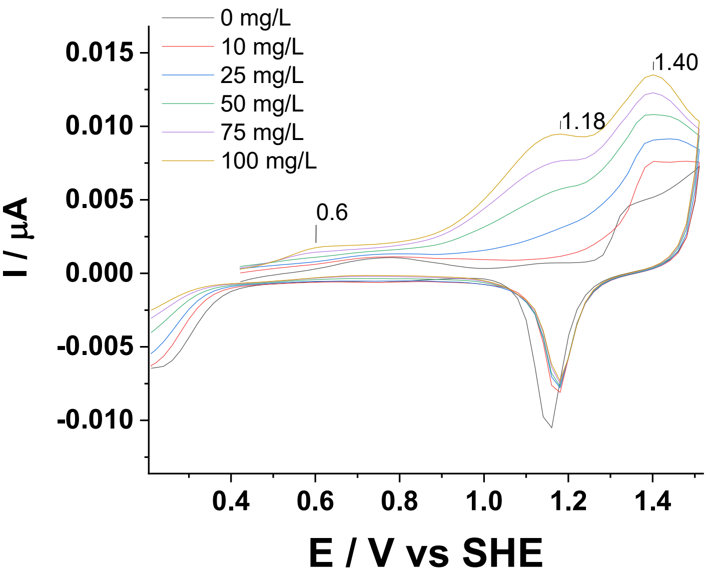

Figure S5: Cyclic voltammogram of MoS_2 (10 mg L^{-1}) on a commercial bare gold electrode at 50 mV s^{-1} in the potential range of -0.4 – 1.4 $V_{Ag/AgCl}$.

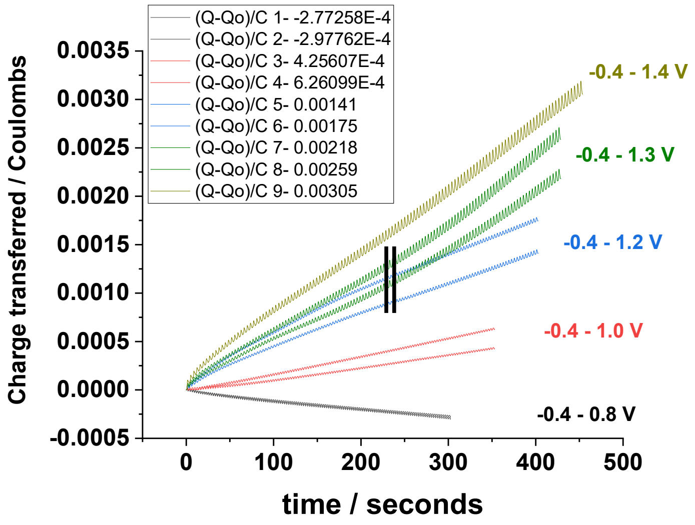

Figure S6: Representative charge transfer curves as a function of potential windows used for the electrodeposition of MoS_2 on a commercial gold electrode.

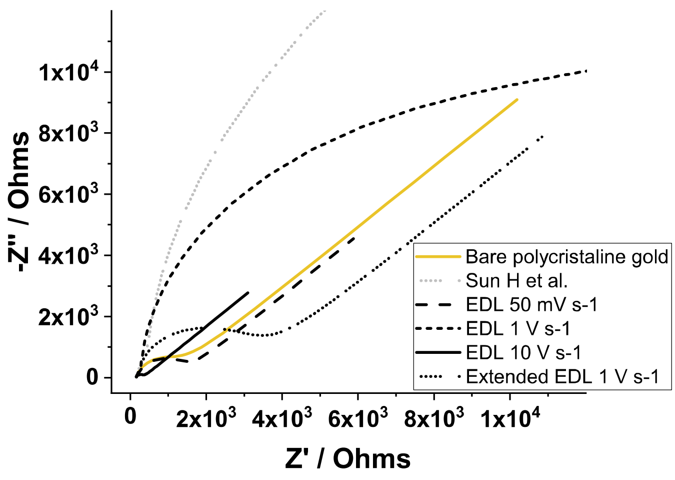

Figure S7: Nyquist plot of the bare and modified electrodes in the presence of ferro/ferricyanide solution (5 mM) in PBS solution pH 7.4. The Plots were fitted with the circuits displayed in Figure S1 and the resulting resistance and capacitance values are summarized in Table S2.

Table S2. The values of the fitted parameters of the EIS analysis. R is in Ohm; C is in Farad.

Sample	R_{sol} [Ohm]	R_{MoS2} [Ohm]	C_{MoS2} [F]	R_{CT} [Ohm]	C_{EDL} [F]	W_{elm} [Ohm $s^{-1/2}$]
Bare	155±5	N/A	N/A	175±55	$0.6±0.1×10^{-6}$	$2.2×10^3$
Ref #20	157±6	333±67	$0.5×10^{-6}$	2600±154	$0.4±0.1×10^{-6}$	$6.3×10^3$
EDL 50mV s^{-1}	155±5	478±43	$0.56×10^{-6}$	750±162	$0.7±0.1×10^{-6}$	$3.6×10^3$
EDL 1V s^{-1}	158±6	521±10	$0.2×10^{-6}$	12900±429	$0.2±0.05×10^{-6}$	$45.9×10^3$
EDL 10V s^{-1}	156±4	1200±23	$0.1×10^{-6}$	24500±625	$0.9±0.1×10^{-6}$	$155×10^3$
Large EDL	157±5	220±510	$0.5×10^{-6}$	820±172	$0.7±0.1×10^{-6}$	$7.2×10^3$

Table S3: Linear regression analysis of the calibration curve of hydroxyurea at the gold (A) and the MoS_2 (B) modified electrodes. The Limit of detection (LoD) correspond to 3 times the intercept error divided by the slope. The LoD error is the intercept error divided by the slope. The sensitivity corresponds to the slope and the sensitivity error to the slope error. The limit of quantification (LoQ) corresponds to 10 times the intercept error.

A)

E / $V_{Ag/AgCl}$	R^2	LoD / µM	LoD Error	Sensitivity / mA cm^{-2} M^{-1}	Sensitivity Error	LoQ / µM
-0.2	0.46816	-310.32934	-103.44311	1.67	0.66669	-930.98802
-0.15	0.33685	-385.09091	-128.36364	1.43	0.7084	-1155.27273
-0.1	-0.07718	-1029.52841	-343.17614	0.66316	0.87828	-3088.58522
-0.05	0.39773	-348.97478	-116.32493	0.89766	0.40297	-1046.92433
0	0.16294	527.93532	175.97844	0.40818	0.27722	1583.80597
0.05	**0.96171**	63.12445	21.04148	18.08	1.47	189.37334
0.1	**0.99183**	28.78543	9.59514	44.88	1.66	86.35628
0.15	**0.99262**	27.34328	9.11443	38.19	1.34	82.02985
0.2	0.84098	135.9126	45.3042	11.67	2.04	407.73779
0.25	-0.1941	4944.95826	1648.31942	0.34437	2.19	14834.8748
0.3	0.17934	-510.94133	-170.31378	3.92	2.58	-1532.82398
0.35	0.60555	-243.19319	-81.0644	10.87	3.4	-729.57958
0.4	0.68619	-206.80625	-68.93542	14.71	3.91	-620.41876
0.45	0.65919	-218.94114	-72.98038	14.27	4.02	-656.82341
0.5	0.5493	-269.60102	-89.86701	11.73	4.07	-808.80307
0.55	0.17025	-520.42171	-173.4739	4.79	3.21	-1561.26514
0.6	0.6533	221.65813	73.88604	7.81	2.23	664.97439

B)

E / $V_{Ag/AgCl}$	R^2	LoD / µM	LoD Error	Sensitivity / mA cm^{-2} M^{-1}	Sensitivity Error	LoQ / µM
-0.2	0.85846	127.13728	42.37909	35.11	5.74	381.41185
-0.15	**0.94195**	78.37827	26.12609	27.52	2.77	235.13481
-0.1	**0.9263**	88.94388	29.64796	15.68	1.79	266.83163
-0.05	0.81049	150.59691	50.19897	9.7	1.88	451.79072
0	0.8445	134.14615	44.71538	10.4	1.79	402.43846

0.05	**0.99734**	16.37612	5.45871	22.28	0.46937	**49.12837**
0.1	**0.99912**	9.42422	3.14141	33.52	0.40639	**28.27267**
0.15	**0.9858**	38.04852	12.68284	28.03	1.37	114.14556
0.2	0.84339	134.67677	44.89226	18.47	3.2	404.03032
0.25	0.79106	159.59257	53.19752	17.77	3.65	478.77772
0.3	0.75806	174.73861	58.2462	15.15	3.41	524.21584
0.35	0.37861	360.15	120.05	8.6	3.98	1080.45
0.4	0.25018	448.4012	149.46707	8.35	4.82	1345.20359
0.45	0.56578	261.73329	87.24443	15.26	5.14	785.19987
0.5	0.86196	125.3408	41.78027	26.35	4.25	376.02239
0.55	**0.98139**	43.63141	14.5438	35.27	1.98	130.89424
0.6	**0.97961**	45.70995	15.23665	36.89	2.17	137.12985

Table S4: Linear regression analysis of the calibration curve for uric acid and ascorbic acid at the gold electrode and the MoS_2 modified electrode. The table correspond to (A) uric acid at gold, (B) ascorbic acid at gold, (C) uric acid at MoS_2 and (D) ascorbic acid at MoS_2.

A)

E / $V_{Ag/AgCl}$	R^2	LoD / µM	LoD Error	Sensitivity / mA cm^{-2} M^{-1}	Sensitivity Error	LoQ / µM
-0.2	0.67862	239.06557	79.68852	42.7	13.89	717.19672
-0.15	0.65113	252.55842	84.18614	20.2	6.94	757.67525
-0.1	0.76094	198.23387	66.07796	7.44	2.01	594.70161
-0.05	0.85815	146.26271	48.75424	3.54	0.70458	438.78814
0	**0.94985**	83.74725	27.91575	2.73	0.31112	251.24176
0.05	**0.91287**	112.34021	37.44674	2.91	0.44485	337.02062
0.1	0.87249	137.9469	45.9823	3.39	0.63638	413.84071
0.15	0.71093	223.46547	74.48849	3.91	1.19	670.39642
0.2	0.79992	177.98837	59.32946	2.58	0.62492	533.96512
0.25	0.7139	222.40714	74.13571	1.4	0.42372	667.22143
0.3	**0.937**	94.53909	31.51303	7.29	0.93786	283.61728
0.35	**0.97392**	59.90909	19.9697	20.13	1.64	179.72727
0.4	**0.982**	49.6342	16.54473	39.01	2.63	148.90259
0.45	**0.98376**	47.10724	15.70241	49.33	3.16	141.32171
0.5	**0.97659**	56.71617	18.90539	38.58	2.98	170.14852
0.55	**0.93149**	98.75465	32.91822	23.11	3.11	296.26395

0.6	0.86419	142.83465	47.61155	12.64	2.46	428.50396

B)

$E / V_{Ag/AgCl}$	R^2	LoD / µM	LoD Error	Sensitivity / mA cm^{-2} M^{-1}	Sensitivity Error	LoQ / µM
-0.2	--	--	--	--	--	--
-0.15	-0.22976	1769.99436	589.99812	0.57461	2.24	5309.98308
-0.1	-0.24273	2969.68797	989.89599	0.14143	0.92479	8909.06391
-0.05	0.10655	359.44182	119.81394	0.65635	0.5195	1078.32547
0	-0.24861	6800.3858	2266.79527	0.04023	0.60236	20401.1574
0.05	0.48466	190.61157	63.53719	1.21	0.50782	571.83471
0.1	**0.97544**	32.19188	10.73063	2.71	0.19207	96.57565
0.15	**0.97633**	31.54558	10.51519	7.24	0.5029	94.63674
0.2	**0.98958**	20.81335	6.93778	11.09	0.50827	62.44004
0.25	**0.96667**	37.58688	12.52896	14.33	1.19	112.76064
0.3	**0.96017**	41.19439	13.73146	18.88	1.71	123.58316
0.35	**0.95228**	45.23045	15.07682	21.61	2.15	135.69135
0.4	**0.91286**	62.16359	20.7212	17.36	2.38	186.49078
0.45	0.88289	73.04061	24.34687	11.82	1.9	219.12183
0.5	0.79084	101.72321	33.90774	6.72	1.51	305.16964
0.55	0.37875	225.29302	75.09767	2.15	1.07	675.87907
0.6	0.50222	184.59502	61.53167	4.42	1.8	553.78507

C)

$E / V_{Ag/AgCl}$	R^2	LoD / µM	LoD Error	Sensitivity / mA cm^{-2} M^{-1}	Sensitivity Error	LoQ / µM
-0.2	0.25434	478.59048	159.53016	3.15	2.05	1435.77143
-0.15	**0.94123**	91.18009	30.39336	2.11	0.26182	273.54028
-0.1	**0.9257**	102.96053	34.32018	2.28	0.31945	308.88158
-0.05	0.81531	170.22857	56.74286	2.1	0.48645	510.68571
0	0.73271	212.10471	70.70157	1.91	0.5513	636.31414
0.05	**0.95372**	80.52632	26.84211	1.9	0.2082	241.57895
0.1	0.78386	187.02809	62.3427	1.78	0.45303	561.08427
0.15	**0.96862**	65.86535	21.95512	5.57	0.49924	197.59605
0.2	0.89884	121.55592	40.51864	42.65	7.06	364.66776
0.25	**0.99001**	36.87288	12.29096	93.38	4.69	110.61865

0.3	**0.98001**	52.35138	17.45046	57.63	4.11	157.05414
0.35	**0.94375**	89.05997	29.68666	25.18	3.05	267.1799
0.4	**0.95709**	77.35602	25.78534	13.37	1.41	232.06806
0.45	**0.92944**	100.24555	33.41518	8.43	1.15	300.73665
0.5	**0.98345**	47.54336	15.84779	5.65	0.36555	142.63009
0.55	-0.33224	14793.3931	4931.13102	0.05526	1.11	44380.1792
0.6	0.51871	318.78777	106.26259	5.56	2.41	956.36331

D)

E / $V_{Ag/AgCl}$	R^2	LoD / μM	LoD Error	Sensitivity / mA cm^{-2} M^{-1}	Sensitivity Error	LoQ / μM
-0.2	0.87745	74.8456	24.94853	15.35	2.53	224.53681
-0.15	**0.98431**	25.58848	8.52949	7.29	0.41077	76.76543
-0.1	**0.93314**	53.93213	17.97738	4.42	0.5249	161.79638
-0.05	**0.99354**	16.375	5.45833	4.56	0.16445	49.125
0	**0.9726**	34.00406	11.33469	4.93	0.36915	102.01217
0.05	**0.92945**	**55.53552**	18.51184	5.49	0.67138	166.60656
0.1	**0.9564**	43.15768	14.38589	9.64	0.91611	129.47303
0.15	**0.95848**	42.08465	14.02822	14.53	1.35	126.25396
0.2	**0.96409**	39.05996	13.01999	17.01	1.46	117.17989
0.25	**0.97874**	29.87448	9.95816	19.36	1.27	89.62345
0.3	**0.99462**	14.92832	4.97611	20.09	0.66038	44.78497
0.35	**0.99688**	11.36834	3.78945	20.66	0.51717	34.10503
0.4	**0.99946**	4.70553	1.56851	20.07	0.20794	14.11659
0.45	**0.99138**	18.91662	6.30554	18.23	0.75931	56.74986
0.5	**0.98998**	20.40625	6.80208	16.32	0.73333	61.21875
0.55	**0.96631**	37.77857	12.59286	12.6	1.05	113.33571
0.6	**0.9147**	61.43333	20.47778	9	1.22	184.3

Table S5: Table depicting the important physicochemical parameters of the organic molecules used in the study.

Compound	Cha-rge	pKa	Mw [g mol^{-1}]	Water solubility	H-bonds (D/A)	Predicted LogP	E_{peak} measured with gold [$V_{Ag/AgCl}$]	E_{peak} measured with MoS_2 [$V_{Ag/AgCl}$]
Hydroxyurea	0	10.14	76.05	269	2NH+1OH/2O	-1.6	0.1	-0.2 (-0.3)
Uric acid	0/-1	5.6	168.11	0.06	3NH/2O	-2.17	0.45	0.25 (-0.2)
L-ascorbic acid	-1	4.2	176.12	245	4OH/5O	-1.85	0.35	0.35

Table S6: Comparisons with literature. We compare the limit of detection and sensitivity of the MoS_2 modified electrode toward hydroxyurea detection (Table S6A) to different electrode architecture. We compare the limit of detection and sensitivity of the MoS_2 modified electrode toward L-ascorbic acid and uric acid (Table S6B) with electrode architecture incorporating MoS_2 as a component.

Table S6A				
Reference	Material	LoD [µM]	LoD [µM] (IUPAC)	Sensitivity [mA cm^{-2} M^{-1}]
This work	Gold		28+/-9	45
This work	MoS_2		9+/-3	34
[3]	Gold	0.92	207	1091
[3]	Glassy carbon	0.46	164	1126
[4]	Au/AgNRs-MIP	0.007	0.007	38
[4]	Pencil graphite	7.89	37	8.9
[5]	Carbon paste	6.52	60	3

Table S6B		L-ascorbic acid			Uric acid		
Reference	Material	LoD [µM]	Sensitivity [mA cm^{-2} M^{-1}]	E [$V_{Ag/AgCl}$]	LoD [µM]	Sensitivity [mA cm^{-2} M^{-1}]	E [$V_{Ag/AgCl}$]
This work	Gold	20 +/-7	20	0.35	11+/-2	49	0.45

This work	MoS_2	4 +/-1	20	0.35	45+/-11	94	0.25
[2]	MoS_2/gold nanoparticles	4870	1091		420	1126	
[6]	MoS_2/Glassy carbon				13.91	12	
[7]	MoS_2/Glassy carbon	5.83	120		0.95	10517	

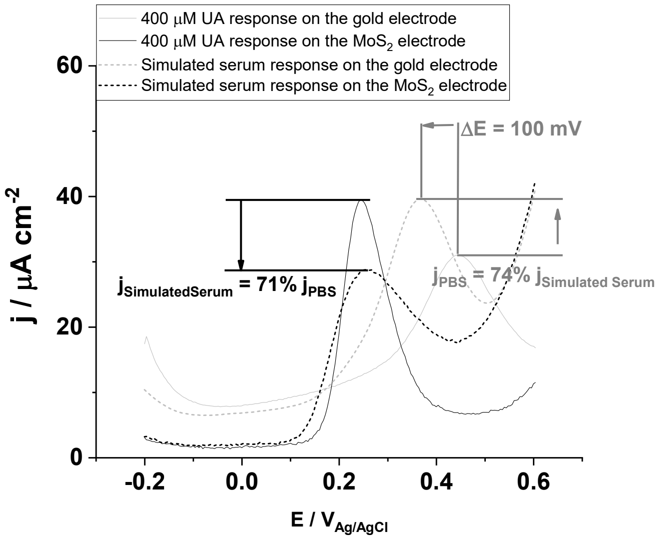

Figure S8: Superposition of the electrochemical signature of uric acid in PBS (plain traces) and in the simulated serum (dashed traces). The oxidation peak shift of 100 mV toward cathodic potential for the gold electrode (grey traces) is associated with an increase of oxidation current from 2.2 to 2.8 µA. The decrease of current of 71% for the MoS_2 electrode (black traces) is associated with the loss of selectivity in the presence of >28 redox active interfering species.

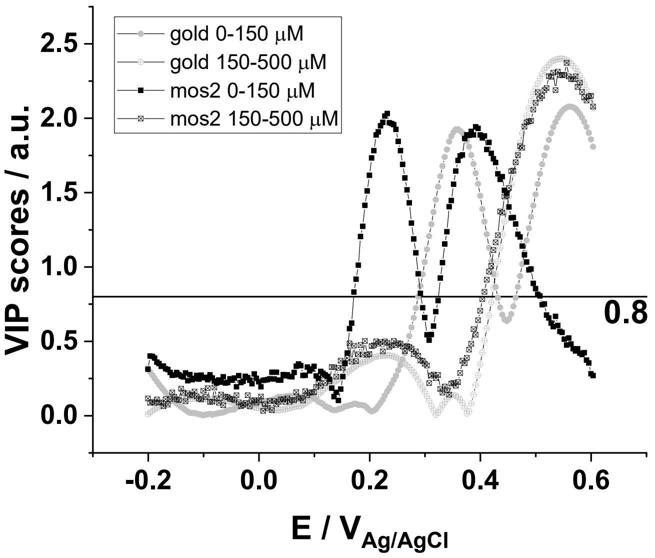

Figure S9: Variable importance plot (VIP) for low (0-150 μM), high (150-500 μM) hydroxyurea concentrations. The bare gold electrode in grey and the MoS_2 modified electrode in black.

Table S7: Limit of detection, taken as 3 time the intercept error of the linear regression analysis of the predicted vs actual hydroxyurea concentration, resulting from the PLSR analysis in PBS (A) and in the simulates serum (B).

A)	Dataset	LoD / μA	LoD Error	R^2	PRESS / μA	# of variables
	Gold - 0-150	6.68274	2.22758	0.99886	6.45	1
	Gold - 150-500	20.83329	6.94443	0.99916	25.6	2
	MoS_2 - 0-150	5.91309	1.97103	0.99971	8.35	3
	MoS_2 - 150-500	7.89399	2.63133	0.99841	26.2	2
	Gold - 0-500	16.38564	5.46188	0.99948	15.8	2
	MoS_2 - 0-500	1.71E-14	5.68E-15	1	13.7	6
	parallel gold	8.28324	2.76108	0.988	18.99	3
	parallel MoS_2	32.15781	10.71927	1.00268	18.99	3
	series	10.0806	3.3602	0.99916	10.57	3

B)	Dataset	LoD / μA	LoD Error	R^2	PRESS / μA	# of variables
	Gold - 0-150	54.054	18.018	0.87892	40	1

Gold - 150-500	61.57059	20.52353	0.98887	25	1
MoS_2 - 0-150	3.63459	1.21153	0.9995	17	2
MoS_2 - 150-500	5.36454	1.78818	0.99992	4	1
Gold - 0-500	16.51992	5.50664	0.99728	21	2
MoS_2 - 0-500	15.0129	5.0043	0.99776	18	2
parallel gold	7.65336	2.55112	0.99941	15	7
parallel MoS_2	5.31414	1.77138	0.99972	15	7
series	5.46687	1.82229	0.9997	12	3

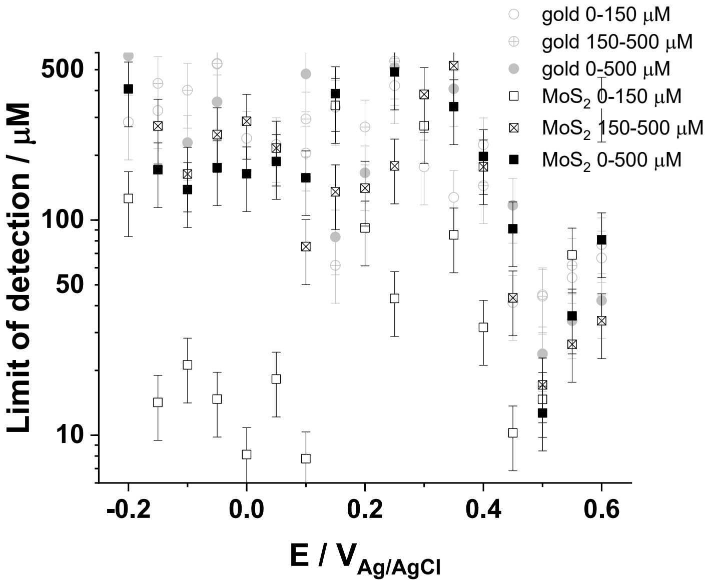

Figure S10: Comparisons of the limit of detection for hydroxyurea taken from linear regression analysis of the calibration curve at each potential for the gold electrode (grey) and the MoS_2 electrode (black) and for different concentration ranges.

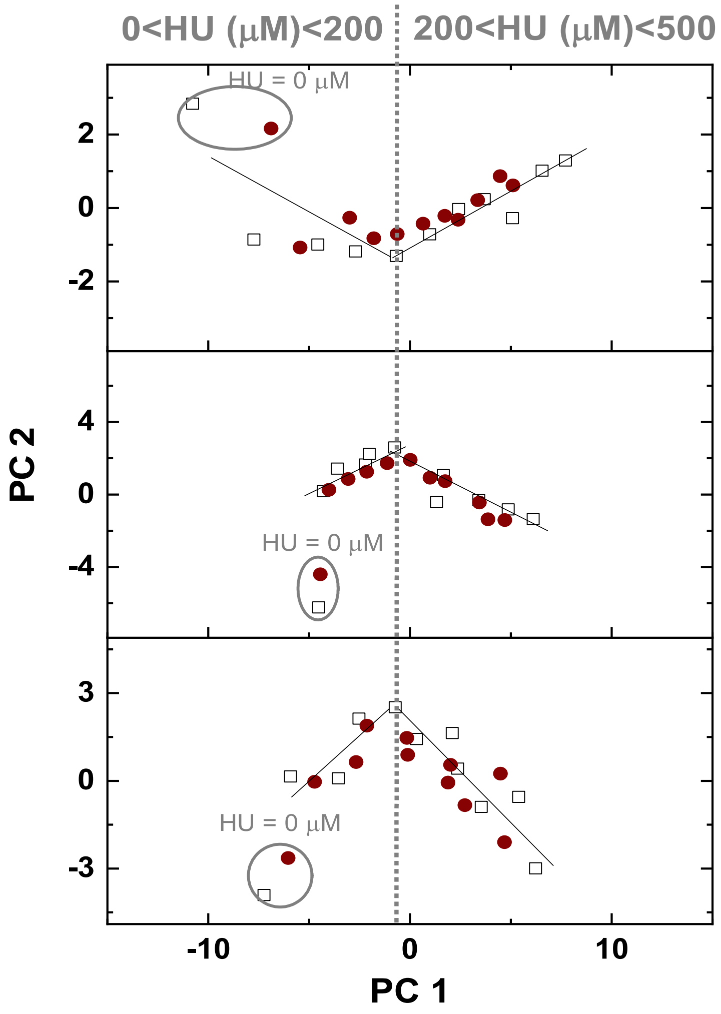

Figure S11: The PCA analysis is used to ensure that the datasets resulting from the data pretreatment step are correlated with their original counterparts.

1. Psychogios, N. *et al.* The human serum metabolome. *PLoS One* **2011,** *6*, e16957.
2. Sun, H. *et al.* Gold nanoparticle-decorated MoS_2 nanosheets for simultaneous detection of ascorbic acid, dopamine and uric acid. *RSC Adv.* **2014,** *4*, 27625–27629.
3. Naik, K. M.; S. T. N. Novel electroanalysis of hydroxyurea at glassy carbon and gold electrode surfaces. *J. Electrochem. Sci. Eng.* **2014,** *4*, 111–121.
4. Pathak, P. K.; Kumar, A.; Prasad, B. B. Functionalized nitrogen doped graphene quantum dots and bimetallic Au/Ag core-shell decorated imprinted polymer for electrochemical sensing of anticancerous hydroxyurea. *Biosens. Bioelectron.* **2019,** *127*, 10–18.
5. Naik, K. M.; Alagur, M. M.; Nandibewoor, S. T. Electrochemical response of hydroxyurea by different voltammetric techniques at carbon paste electrode. *Anal. Methods* **2013,** *5*, 6947–6953.
6. Yan, B. *et al.* Glassy carbon electrode modified with G-MoS_2-Nafion acts as an electrochemical biosensor to determine uric acid in human serum. *Mol. Med. Rep.* **2018,** *18*, 3193–3202.
7. Li, Y., Lin; H., Peng, H.; Qi, R.; Luo, C. A glassy carbon electrode modified with MoS_2 nanosheets and poly(3,4-ethylenedioxythiophene) for simultaneous electrochemical detection of ascorbic acid, dopamine and uric acid. *Microchim. Acta* **2016,** *183*, 2517–2523.
